# A Review of Onychomycosis Due to *Aspergillus* Species

**DOI:** 10.1007/s11046-017-0222-9

**Published:** 2017-11-16

**Authors:** Felix Bongomin, C. R. Batac, Malcolm D. Richardson, David W. Denning

**Affiliations:** 1 The National Aspergillosis Centre, Education and Research Centre, Wythenshawe Hospital, Manchester University NHS Foundation Trust, Southmoor Road, Manchester, M23 9LT UK; 2 Skin Study Group, Institute of Herbal Medicine, National Institutes of Health, University of the Philippines - Manila, Manila, Philippines; 3 NHS Mycology Reference Centre, Wythenshawe Hospital, Manchester University NHS Foundation Trust, Southmoor Road, Manchester, M23 9LT UK; 4 Division of Infection, Immunity and Respiratory Medicine, School of Biological Sciences, Faculty of Biology, Medicine and Health, The University of Manchester, Oxford Road, Manchester, M13 9PL UK

**Keywords:** *Aspergillus*, Onychomycosis, Clinical features, Epidemiology, Mycology

## Abstract

*Aspergillus* spp. are emerging causative agents of non-dermatophyte mould onychomycosis (NDMO). New *Aspergillus* spp. have recently been described to cause nail infections. The following criteria are required to diagnose onychomycosis due to *Aspergillus* spp.: (1) positive direct microscopy and (2) repeated culture or molecular detection of *Aspergillus* spp., provided no dermatophyte was isolated. A review of 42 epidemiological studies showed that onychomycosis due to *Aspergillus* spp. varies between < 1 and 35% of all cases of onychomycosis in the general population and higher among diabetic populations accounting for up to 71% and the elderly; it is very uncommon among children and adolescence. *Aspergillus* spp. constitutes 7.7–100% of the proportion of NDMO. The toenails are involved 25 times more frequently than fingernails. *A. flavus*, *A. terreus* and *A. niger* are the most common aetiologic species; other rare and emerging species described include *A. tubingensis*, *A. sydowii*, *A. alliaceus*, *A. candidus*, *A. versicolor*, *A. unguis*, *A. persii, A. sclerotiorum, A. uvarum, A. melleus, A. tamarii* and *A. nomius*. The clinical presentation of onychomycosis due to *Aspergillus* spp. is non-specific but commonly distal–lateral pattern of onychomycosis. A negative culture with a positive KOH may point to a NDM including *Aspergillus* spp., as the causative agent of onychomycosis. Treatment consists of systemic therapy with terbinafine or itraconazole.

## Introduction

Onychomycosis are caused by dermatophytes, non-dermatophyte (saprophytic) moulds (NDMs) or yeasts. *Aspergillus* spp., *Scopulariopsis* spp., *Alternaria* spp., *Acremonium* spp. and *Fusarium* spp. are the most common NDM agents reported to be responsible for approximately 2–25% of all the causes of onychomycoses [1–3]. NDM onychomycosis presents with clinical features mimicking dermatophytic onychomycosis, making clinical diagnosis difficult and unreliable [4]. Very little is known regarding the ability of NDM to invade an intact nail plate. [5]

*Aspergillus* spp. are increasingly being reported as primary causative agents of onychomycosis worldwide with prevalence as high as 34.4% in Guatemala [6], 69.3% in Iran [4] and up to 71% among diabetic patients in Sri Lanka [7]. It has been previously thought that same species of *Aspergillus* were responsible for both superficial (e.g. onychomycosis) and systemic infections; however, many new species of *Aspergillus* are increasingly being reported to cause onychomycosis and these were not previously reported to cause systemic infections [8, 9].

The isolation of *Aspergillus spp.* from nail specimens may mean several things: causative agent, coloniser or contaminant. *Aspergillus spp*. isolated from nail specimens are not susceptible to most of the topical and systemic antifungals used to treat dermatophytes [2]. Resistance to triazole antifungals occurs among the *Aspergilli* [10] and inadequate treatment may lead to resistance and recurrence of infection. Proper clinical diagnosis, laboratory workup and adequate antifungal therapy are thus the standard of care for *Aspergillus* infections.

The prevalence, clinical manifestations and mycological characteristics of onychomycosis caused by *Aspergillus* spp. are poorly understood. This review evaluates the clinicomycological characteristics and epidemiology of onychomycosis due to *Aspergillus*. We use the species name to describe both that species and closely related, often cryptic species, for the commonest species complex.

## Epidemiology of Onychomycosis due to *Aspergillus* Species

Onychomycosis is a common condition accounting for up to 18–50% of all nail diseases and 30% of cutaneous fungal infections [11]. The global burden of fungal nail, skin and hair infections is about 1 billion cases [12], translating to nearly 300 million cases of onychomycosis globally. *Aspergillus* species accounts for 0.5–3% of all cases of onychomycosis [13]; therefore, about 10 million cases of onychomycosis are attributable to *Aspergillus* spp.

We reviewed data from 42 epidemiological studies from 19 countries across the globe between 1974 and 2017. The prevalence of onychomycosis due to *Aspergillus* spp., both as percentage of all causes of onychomycosis and as percentage of NDM, shows a marked geographical variation among countries, different regions of same country, over time in the same region and underlying co-morbid conditions or occupational predisposition (Table 1). As an overall cause of onychomycosis, the prevalence of onychomycosis due to *Aspergillus* spp. varies between < 1 and 35% in the general population and higher among diabetic populations at 71% (Table 1). *Aspergillus* spp. constitutes 7.7–100% of the proportion of NDM onychomycosis. Over 50% (23/42) of the reviewed epidemiological studies reportedly isolated *Aspergillus* spp. in 50–100% of the NDMs. Onychomycosis due to *Aspergillus* spp. is thus more prevalent and emerging cause of onychomycosis than previously thought.

**Table 1:** Prevalence of *Aspergillus* onychomycosis

Author/references	Year	Country	Number of cases*	% of total cause of onychomycosis	% of total non-dermatophyte	Most common *Aspergillus* species	Comments
Moubasher et al. [38]	2017	Assiut, Egypt	125	15.9	19.5	*A. niger*, *A. flavus* and *A. terreus*	–
Martínez-Herrera et al. [6]	2016	Guatemala	32	–	34.4	Not stated	Opportunistic mould onychomycosis
Motamedi et al. [4]	2016	Tehran, Iran	424	12.3	69.3	*A. flavus*	
Chadeganipour et al. [39]	2016	Isfahan, Iran	1,284	9.1	62.2	*A. flavus* (66%)	*A. nidulans* (16%), *A. fumigatus* (10%) *A. terreus* (8%)
Wijesuriya et al. [7]	2015	Sri Lanka	255	71.0	100	*A. niger* (76%)	Diabetic populations
Nouripour-Sisakht et al. [14]	2015	Tehran, Iran	463	29.2	87.7	*A. flavus* (77.3%)	*A. niger* (3%), *A. tubingensis* (3%), *A. terreus* (2.2%), *A. sydowii* (2.2%)
Raghavendra et al. [28]	2015	Rajasthan, India	150	30.0	84.9	*A. flavus* (53.3%)	*A. niger* (33.3) and *A. fumigatus* (13.3%)
Soltani et al. [20]	2015	Tehran, Iran	79	–	50	Not stated	–
Afshar et al. [40]	2014	Mazandaran, Iran	625	14.2	89.3	*A. flavus* (67%)	Toe and finger nails
Shahzad et al. [26]	2014	Qassim, Saudi Arabia	77	29.9	82.1	Not stated	–
Morales-Cardona et al. [41]	2014	Bogota, Colombia	317	–	2.6	Not stated	
Mikaeili et al. [42]	2013	Kermanshah, Iran	1086	2.2	75.0	*A. flavus* (50%),	*A. niger* (33%), *A. fumigatus* (17%)
Vasconcellos et al. [43]	2013	Sao Paolo, Brazil	35	5.6	33.3	Not stated	Institutionalised elderly patients
Dhib et al. [44]	2012	Central, Tunisia	5789	1.1	42.7	*A. flavus* (44.3%), *A. niger* (18%)	A 22-year retrospective study
Hajoui et al. [32]	2012	Morocco	150	–	35.3	Not stated	20-year retrospective study on only mould onychomycosis
Leelavathi et al. [45]	2012	Malaysia	231	35.1	59.8	Not stated	5-year retrospective study
Minkoumou et al. [46]	2012	Cameroon	52	13.5	70.0	*A. niger* (71%)	*A. unguis* (14%), *A. alliaceus* (14%)
Ranawaka et al. [47]	2012	Galle, Sri lanka	128	30.6	66.7	*A. niger* (73.1%),	*A. flavus* (19.2%), *A. terreus* (7.7%)
Aghamirian et al. [48]	2010	Qazvin, Iran	124	3.2	100	*A. niger and A. flavus (50% each)*	All-cause: No other NDM isolated
Bassiri-Jahromi et al. [30]	2010	Tehran, Iran	410	6.8	59.6	*A. fumigatus* (27.6%)	–
Souza et al. [49]	2010	Goiania, Brazil	1282	0.08	50	Not stated	Only 1 patient had *Aspergillus* isolate
Adhikari et al. [50]	2009	Sikkim, India	32	21.43	60	*A. niger* (100%)	–
Godoy et al. [51]	2009	Sao Paolo, Brazil	247	0.6	7.7	Not stated	–
Hashemi et al. [52]	2009	Tehran, Iran	216	9.7	51.2	*A. flavus* (43%)	*A. niger* (19%), *A. fumigatus* (14%).
Chadeganipour et al. [53]	2008	Isfahan, Iran	185	22.2	77.4	*A. flavus* (59%),	*A. nidulans* (12%), *A. fumigatus* (7.3)
Das et al. [54]	2008	Eastern, India	44	18.2	80.0	*A niger* (100%)	Finger nail onychomycoses
Manzano-Gayosso et al.	2008	Mexico	70	1.4	16.7	*A. fumigatus* (100%)	Type 2 diabetes mellitus patients
Surjushe et al. [55]	2007	Mumbai, India	60	5.0	15.8	*A. niger* (100%)	HIV-infected persons
Veer et al. [56]	2007	India	72	14	50.0	Not stated	Of the non-dermatophyte moulds
Gupta et al. [19]	2007	Himachal Pradesh, India	130	6.1	33.3	Not stated	–
Bonifaz et al. [57]	2007	Mexico	5221	0.51	34.6	*A. niger*	Retrospective study 1992–2005
Hilmioglu-Polat et al. [58]	2005	Izmir, Turkey	1,146	1.5	30.3	*A. niger* (70%)	*A. flavus* (10%), *A. fumigatus* (10%), *A. terreus* (10%)
Boukachabine et al. [59]	2005	Morocco	–	–	12.0	–	22-year (1982–2003) retrospective study.
Romano et al. [21]	2005	Italy	46	2.2	33.3	*A. fumigatus* (100%)	In children
Gianni and Romano [27]	2004	Italy	1,228	2.6	47.9	*A. fumigatus* (29%)	*A. niger* (21%), *A. terreus* (12%)
Piraccini et al. [60]	2004	Italy	79	6	29.4	Not stated	Cases of white superficial onychomycosis
Grover et al. [33]	2003	Bangalore and Jorhat, India	50	18.6	84.6	*A. niger* (100%)	–
Romano et al. [61]	2003	Italy	4,046	3.3	25.2	Not stated	15-year survey
Bokhari et al. [62]	1999	Lahore, Pakistan	100	2	18.2	Not stated	
Ramani et al. [63]	1993	Karnataka, India	100	19	86.4	*A. niger* (36.8%)	*A. fumigatus* (31.6%), *A. flavus* (15.8%)
Lim et al. [64]	1992	Singapore	100	3.0	25.0	Not stated	–
English and Atkinson [3]	1974	Bristol, UK	216		75.0	*A. terreus* (53%), *A. versicolor* (40%), *A. nidulans* and *A. candidus* (3% each)	Elderly chiropody patients

*This refers to the number of onychomycosis cases investigated in the study, regardless of cause

Generally, *A. niger* (complex) and *A. flavus* (complex) are the commonest species group of *Aspergillus* isolated from abnormal nail specimens. *A. fumigatus*, *A. terreus* and *A. nidulans* are also common, as reported from the epidemiological studies (Table 1). Rare and newly described species from case reports include *A. tubingensis*, *A. sydowii*, *A. alliaceus*, *A. candidus*, *A. versicolor*, *A. unguis*, *A. persii, A. sclerotiorum, A. uvarum, A. melleus, A. tamarii, A. nomius* and others, some of which are with the main pathogenic species complexes [9, 14].

## Predisposing Factors

Factors such as increasing age, nail trauma, immunodeficiency, hyperhidrosis, socio-economic status, poor hygiene, climatic conditions, occupational exposures such as gardening and house chores, barefoot walking and paronychia predispose to onychomycosis [15]. Furthermore, damage can also be induced by hormonal disturbances (diabetes mellitus, Cushing’s syndrome and hypothyroidism) or by HIV/AIDS immunosuppression or ongoing biological (immunosuppressive) therapies [16]. Among diabetics with onychomycosis in Sri Lanka, onychomycosis due to *Aspergillus* spp. occurred in 71%, among which *A. niger* (76%) and *A. flavus* (12%) were the most predominant species isolated [7]. The same study showed that the risk of having *Aspergillus* onychomycosis among diabetics increased with age and duration of diabetes.

None of the above predisposing factors is specific for *Aspergillus* spp. However, *Aspergillus* onychomycosis is seen more among individuals with occupational exposures such as vegetable vendors [17] and among babassu coconut breakers [18], diabetics and the elderly [19]. Some individuals diagnosed with onychomycosis due to *Aspergillus* spp. do not have identifiable predisposing conditions/occupational risk factors. In fact, Soltani and colleagues in their study reported that up to 70% of patients with *Aspergillus* onychomycosis had no predisposing conditions [20]. Onychomycosis due to *Aspergillus* spp. is very uncommon in children. [21, 22]

## Pathophysiology

*Aspergillus* spp. are ubiquitous environmental moulds found in soil, decaying vegetation and water and are not transmitted from person to person [23]. Infection starts under the nail near the hyponychium where spores may have lodged or at the lateral nail folds, or on a diseased nail plate colonised by *Aspergillus* spp. [24]. Once the fungus starts to grow, the infection spreads back towards the cuticle. It looks much the same as any fungal nail infection, discolouring the nail, causing it to become thick, distorted and flaky [25]. An early experimental study with *A. versicolor* using healthy nail samples showed that *A. versicolor* could only grow on the surface of the nail without penetrating the nail plate [5]. An evidence of the non-keratinolytic potentials of these moulds. *Aspergillus* species growing in nature often produces colourful pigments; therefore, an *Aspergillus* nail infection may well appear greenish, black, brown or various other shades [17]. The fungus will not, however, spread to the surrounding skin like some other fungal causes of nail infection [17].

## Clinical Manifestation

Onychomycosis due to *Aspergillus* spp. is usually a distal–lateral subungual onychomycosis (DLSO). The toenails are involved 25 times more frequently than fingernails due to increased exposure to soil, water and decaying vegetation where *Aspergillus* moulds thrive [17].

**Fig. 1:**
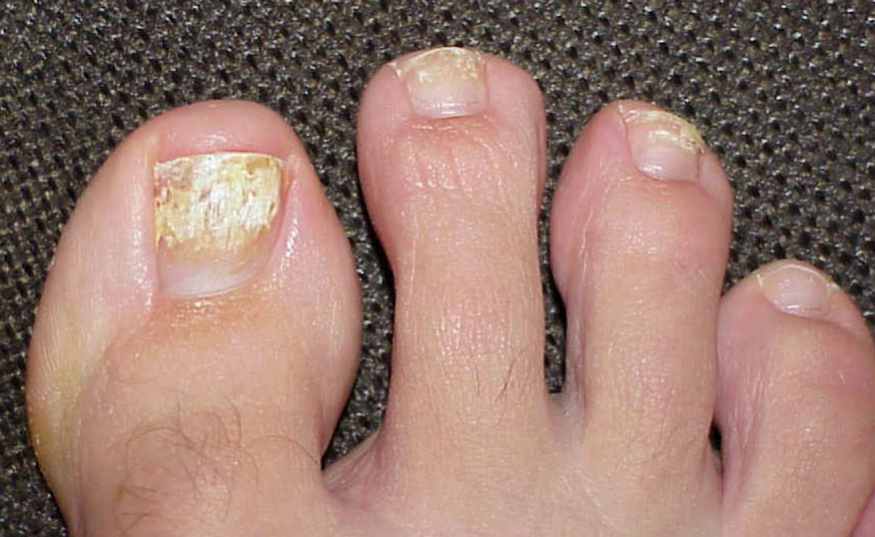
Distal–lateral subungual onychomycosis caused by *Aspergillus terreus* in a 60-year-old immunocompetent man. A flaky, whitish, sharply demarcated patch surrounded by a yellowish discoloration is noted on the distal 2/3 of the first toenail. Similar lesions are noted on the second and third toenails. The first toenail shows signs of paronychia with beginning erythema and swelling of the distal and lateral nail folds. Note the SWO component especially on the first toenail (Courtesy of Prof. David W. Denning, the National Aspergillosis Centre, Manchester, UK)

Few studies have been carried out correlating the clinical classification with the causative fungal species. One of such studies from Saudi Arabia showed that 17 of the 23 (74%) cases of onychomycosis due to *Aspergillus* spp. were DLSO, 5/23 (22%) were proximal subungual onychomycosis (PSO), 2/23 (9%) were superficial white onychomycosis (SWO) and 1/23 (4%) was total dystrophic onychomycosis (TDO) [26]. In a systemic review of NDM onychomycosis, Gupta and colleagues reported that *Aspergillus* spp. manifest as PSO in 37.5% of the cases, DLSO in 26.1% and SWO in 25.5% [2]. A study in Italy showed that the clinical features suggesting onychomycosis due to *Aspergillus* spp. are (1) chalky deep white nail, (2) rapid involvement of lamina and (3) painful perionyxis without pus [27]. The clinical pattern, however, appears to vary depending on the *Aspergillus* species implicated. Raghavendra and colleagues in India described *A. flavus* causing 19.2% of DLSO, 18.8% of TDO and 9.1% SWO. In contrast, *A. niger* was associated with 11.5% of DLSO, 10.1% of TDO, 9.1% of SWO and 6.3% of mixed pattern onychomycosis (MPO), whereas *A. fumigatus* was associated with DLSO in 2% of the patients, 5.8% TDO and 6.3% MPO [28]. In *A. terreus* onychomycosis (Fig. 1), the observed clinical patterns in fingernail were DLSO (33.3%), SWO (33.3%) and onycholysis (33.3%), and in toenail, SWO (52.9%) was the most frequent clinical pattern followed by DLSO (42.0%) and DLSO plus SWO (5.9%) [29]. Another study showed that among those with onychomycosis due to *Aspergillus* spp., 93% manifested with hard nails, 89% with brittle nails and 85% had discoloured nails. Involvement of surrounding skin is not common [17]. Subungual hyperkeratosis is almost always present in all patients with mould onychomycosis regardless of the genus of the fungi isolated [30]. Onychomycosis due to *Aspergillus* spp. are sometimes associated with subungual dermatophytoma (“fungal ball”) formation. [6]

## Diagnosis

NDM onychomycosis and therefore onychomycosis due to *Aspergillus* spp. may be considered in patients with fungal infection in a diseased or traumatised nail, without associated skin involvement, unresponsive to commonly used antifungal agents [31]. It may be suspected in cases which were KOH positive and culture negative for dermatophytes [31].

The differential diagnoses for onychomycosis due to *Aspergillus* spp. are very broad and include yeast nail infections, tinea unguium, non-*Aspergillus* spp. NDMO and other non-fungal nail infections and disorders. Therefore, the diagnosis of onychomycosis due to *Aspergillus* spp. is both clinical and mycological. Since there are no specific signs associated with onychomycosis due to *Aspergillus* spp., it is not possible to diagnose it based solely on physical appearance.

Determining the mycological cause of onychomycosis is helpful in guiding antifungal treatment and preventing complications [32]. Identification of the fungal agent directs the treatment plan, as well as prognosis. Since culture of NDMs from nail specimens does not always translate to causation, Gupta et al. [2] proposed that at least 3 of the following criteria should be satisfied: (1) KOH positive, (2) culture of NDM, (3) repeated culture (2–3) of NDMs, provided no dermatophyte was isolated, (4) histopathology using periodic acid–Schiff staining positive for fungal elements, (5) culture of NDM from 5 of 20 nail inoculated nail fragments and (6) NDM identification through molecular techniques. However, a positive direct microscopy, and repeated culture or molecular detection of *Aspergillus* spp., provided no dermatophyte was isolated is sufficient to diagnose *Aspergillus* onychomycosis. Aspergillary heads may be observed with direct microscopic examination of nail specimens, especially in very chronic cases or with onycholysis. [1]

Mycological culture on Sabouraud’s dextrose agar with or without cycloheximide yields fungal isolates in less than 50% of the cases. However, combining KOH preparation and culture, sensitivity is increased to 85.8%. [33] Isolation rate is higher (83%) for nail samples obtained by drilling compared to scraping (67%) [34].

## Treatment

Treatment options for NDM onychomycosis are still limited; however, onychomycosis caused by *Aspergillus* spp. responds well to systemic antifungal agents, with itraconazole performing better than terbinafine in vitro [31]. Affected fingernails typically require 3-month therapy and toenails at least 6 months. Terbinafine given as pulse (500 mg per day for 1 week every month for 3 months) produced complete cure in 30 of 34 cases on the 12-month follow-up [2]. Tosti et al. recommend either daily terbinafine (250 mg per day) or pulse itraconazole (400 mg per day for 1 week per month) for 2–4 months, completely curing 5 of 5 patients with *Aspergillus* onychomycosis who accepted treatment [35]. Interestingly, the nail discolorations in *Aspergillus* onychomycosis often persist despite evidence of mycological cure [17]. Systemic antifungals are best combined with chemical nail avulsion using 40% urea ointment for hyperkeratotic nails and topical ciclopirox olamine nail lacquers for SWO [13]. Terbinafine resistance has been reported with *A. candidus* onychomycosis, and mycological cure was achieved following 10 weeks of itraconazole therapy [36]. Total nail avulsion followed by topical antifungal post-operatively has also been shown to be an effective management option (clinical cure rate 88% and mycological cure rate 100%) for patients with single or oligo-onychomycosis [37]. However, it should be noted that comparative clinical trials on the treatment of *Aspergillus* onychomycosis have not been done to date and that recommendations have been based on case studies.

## Conclusion

Onychomycosis due to *Aspergillus* spp. is more prevalent and emerging cause of onychomycosis than previously thought. The prevalence ranges from 7.7 to 100% of all NDMO and between < 1 and 35% in the general population. Since the clinical presentation of onychomycosis due to *Aspergillus spp.* is non-specific, it is necessary to perform laboratory procedures such as KOH, culture, histopathology and molecular techniques to diagnose it. A positive direct microscopy, and repeated culture or molecular detection of *Aspergillus* spp., provided no dermatophyte was isolated is the required criteria for the diagnosis of onychomycosis due to *Aspergillus* spp. Treatment consists of systemic therapy with terbinafine or itraconazole.

## Abbreviations

DLSO: Distal–lateral subungual onychomycosis
EO: Endonyx onychomycosis
KOH: Potassium hydroxide
MPO: Mixed pattern onychomycosis
NDM: Non-dermatophyte mould
NDMO: Non-dermatophyte mould onychomycosis
PSO: Proximal subungual onychomycosis
SWO: Superficial white onychomycosis
TDO: Total dystrophic onychomycosis
